# Model Evaluation of the Microbial Metabolic Processes in a Hydrogen-Based Membrane Biofilm Reactor for Simultaneous Bromate and Nitrate Reduction

**DOI:** 10.3390/membranes12080774

**Published:** 2022-08-11

**Authors:** Minmin Jiang, Yuanyuan Zhang, Jie Zhang, Xingru Dai, Haixiang Li, Xuehong Zhang, Zhichao Wu, Junjian Zheng

**Affiliations:** 1 Guangxi Key Laboratory of Environmental Pollution Control Theory and Technology, Guilin University of Technology, 319 Yanshan Street, Guilin 541006, China; 2 College of Life and Environmental Science, Guilin University of Electronic Technology, 1 Jinji Road, Guilin 541004, China; 3 School of Chemistry and Materials Engineering, Huizhou University, 46 Yanda Road, Huizhou 516007, China; 4 State Key Laboratory of Pollution Control and Resource Reuse, School of Environmental Science and Engineering, Tongji University, 1239 Siping Road, Shanghai 200092, China

**Keywords:** BrO_3_^−^, NO_3_^−^, bioreactor, microbial reduction, multispecies model, biofilm characteristics

## Abstract

The H_2_-based membrane biofilm reactor (H_2_-MBfR) has been acknowledged as a cost-effective microbial reduction technology for oxyanion removal from drinking water sources, but it remains unknown how the evolution of biofilm characteristics responds to the changing critical operating parameters of the H_2_-MBfR for simultaneous bromate (BrO_3_^−^) and nitrate (NO_3_^−^) elimination. Therefore, an expanded multispecies model, applicable to mechanistically interpret the bromate-reducing bacteria (BRB)- and denitrifying bacteria (DNB)-dominated metabolic processes in the biofilm of the H_2_-MBfR, was developed in this study. The model outputs indicate that (1) increased BrO_3_^−^ loading facilitated the metabolism of BRB by increasing BRB fraction and BrO_3_^−^ gradients in the biofilm, but had a marginal influence on NO_3_^−^ reduction; (2) H_2_ pressure of 0.04 MPa enabled the minimal loss of H_2_ and the extension of the active region of BRB and DNB in the biofilm; (3) once the influent NO_3_^−^ concentration was beyond 10 mg N/L, the fraction and activity of BRB significantly declined; (4) BRB was more tolerant than DNB for the acidic aquatic environment incurred by the CO_2_ pressure over 0.02 MPa. The results corroborate that the degree of microbial competition for substrates and space in the biofilm was dependent on system operating parameters.

## 1. Introduction

Bromate (BrO_3_^−^), a common disinfection byproduct formed during the advanced oxidation processes (especially ozonation) of drinking water, has been assigned as a Group 2B carcinogen with a maximum contaminant level of 10 μg/L in potable water by the World Health Organization [1,2,3]. The BrO_3_^−^ concentration varies typically from below 30 μg/L in the ozonated surface water to 0.8–1.4 mg/L in the groundwater intruded by industrial discharges [2,4]. Current physicochemical approaches for aquatic BrO_3_^−^ elimination, involving adsorption, separation, chemical reduction, and photo-/electro-catalysis, exhibit limited application potential due to their disadvantages in terms of high energy consumption, stringent optional conditions, and requirements of post-treatment steps [5,6,7]. Comparably, autotrophic microbial reduction has received increasing interest in the past decade, owing to its proven capacity of cost-efficiently converting BrO_3_^−^ to innocuous Br^−^ under mild circumstances and the organic-deficient nature of most drinking water sources [6,8].

Nitrate (NO_3_^−^) is a pervasive contaminant which coexists with BrO_3_^−^ at a typical concentration range of 10–30 mg N/L in surface water and groundwater, and which can result in methemoglobinemia in infants [8,9,10,11]. This accentuates the necessity to develop robust technology capable of simultaneously eliminating BrO_3_^−^ and NO_3_^−^ from polluted drinking water sources. As an advanced autotrophic microbial reduction technology, the H_2_-based membrane biofilm reactor (H_2_-MBfR) has been successfully practiced at a pilot and/or commercial scale for eradicating a variety of oxidized pollutants, e.g., BrO_3_^−^, NO_3_^−^, sulfate, perchlorate, selenate, and chromate [9,12]. In this system, electron donor H_2_ diffuses through the wall of gas-permeable hollow fiber membranes (HFM), and supports the functional microorganisms in the HFM-attached biofilm to respire the electron acceptors (i.e., oxidized pollutants); such a scheme of passive gas delivery enables the full utilization of H_2_, as well as the efficient removal of targeted contaminants [9,13]. Several case studies have shown that, when the influent concentrations of BrO_3_^−^ and NO_3_^−^ were in the range of 0.1–1.5 mg/L and 5–20 mg N/L, respectively, which were approachable to those in real drinking water sources, the appreciable removal (beyond 95%) of both oxyanions could be realized in H_2_-MBfRs [1,8].

In H_2_-MBfRs, the biofilm microorganisms, which can dominate the reduction of BrO_3_^−^ and NO_3_^−^, are defined as bromate-reducing bacteria (BRB) and denitrifying bacteria (DNB), respectively [1,11]. Since H_2_ is shared by functional bacteria (e.g., BRB and DNB) in the biofilm as the sole electron donor to drive the reduction of oxidized pollutants, the microbial competition for H_2_ availability (controlled by H_2_ supplying pressure) inevitably occurs in H_2_-MBfRs [14]. The results of empirical studies corroborated that BrO_3_^−^ and NO_3_^-^ concentrations were critical to the efficiencies of microbial reduction, and the BrO_3_^−^ reduction processes could be inhibited in the case of exorbitant NO_3_^−^ loading [11,15]. Microbial community investigations have revealed the variation trends in the species and abundance of BRB and DNB in the biofilm systems under diverse BrO_3_^−^ and NO_3_^−^ loadings [6,11]. Despite the above progress, it remains understudied the extent to which the critical operating parameters drive the evolution of biofilm microenvironment, structure, and activity of the H_2_-MBfR for simultaneous BrO_3_^−^ and NO_3_^−^ elimination. This constitutes a major hindrance to our understanding of the interaction mechanism between BRB and DNB in the biofilm. The development of a systematic and quantitative framework might be a solution to this concern, given the difficulty in the use of present experimental means for elucidating the stratification features of biofilm.

The one-dimensional, multispecies biofilm model, developed on the AQUASIM platform, is a versatile tool applicable to quantitatively assess the steady-state characteristics of the biofilm, e.g., the substrate profiles, as well as microbial distribution and activities [16]. To date, a model framework, which can be implemented to simulate the simultaneous removal processes of BrO_3_^−^ and NO_3_^−^ in the H_2_-MBfR, is still unavailable in the literature. In this work, a sophisticated biofilm model was established on the AQUASIM platform to mechanistically interpret the BrO_3_^−^ and NO_3_^−^ reduction processes of H_2_-MBfR. On the basis of the previous model frameworks for NO_3_^−^ removal [14,16], we proposed an expanded model framework through incorporation into the algorithm for the metabolic process kinetics of BRB, as well as the influences of CO_2_ addition (as carbon source and pH regulator) on metabolic process kinetics of BRB and DNB. Long- and short-term experimental data regarding BrO_3_^−^ and NO_3_^−^ removal, obtained from a lab-scale H_2_-MBfR, were used for the calibration of key model parameters and the validation of the model outputs, respectively. The validated model was then applied to evaluate the gradients of substrates (H_2_, BrO_3_^−^, and NO_3_^−^), as well as the distribution and activities of BRB and DNB in the biofilm, in the case of changing system operating parameters including the H_2_ pressure, CO_2_ addition, and influent BrO_3_^−^ and NO_3_^−^ concentrations.

## 2. Materials and Methods

### 2.1. Reactor Setup and Start-Up

**Figure 1:**
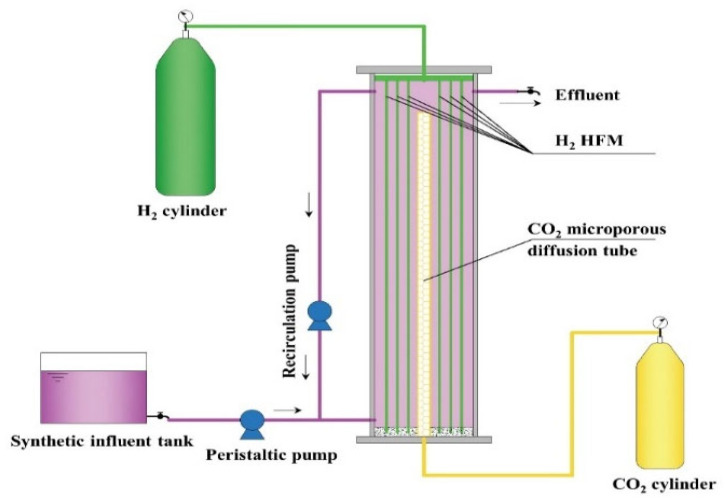
Schematic of the H_2_-MBfR.

The schematic of the H_2_-MBfR used in this study is shown in Figure 1. The reactor consisted of a module containing 96 HFMs (Litree Company, Suzhou, China) and a microporous tube placed in a plexiglass cylinder (height = 22 cm, inner diameter = 6 cm, effective volume = 0.56 L). The average outer diameter and pore size of HFM was 1.5 mm and 0.02 μm, respectively, and the total membrane surface area of HFM module was 633 cm^2^. The module with a sealed lower end was connected to a H_2_ tank for delivering pressurized H_2_. Pure CO_2_ was supplied by the microporous tube with its bottom end linked to a CO_2_ tank to serve as carbon source and pH regulator. Synthetic influent was introduced into the reactor via a peristaltic pump (BT101L-DG-1, Lead Fluid, Baoding, China), and the effluent was collected from the outlet of the reactor. A recirculation pump (BT101L-YZ15/25, Lead Fluid, Baoding, China) was sustained at a high flowrate of 100 mL/min to keep the bulk liquid completely mixed. The reactor was inoculated with 50 mL of biomass collected from a denitrifying H_2_-MBfR in our lab. The synthetic influent, containing 10 mg N/L NaNO_3_ with an identical composition to that described in Li et al. [17], was used in the start-up period of the reactor. During the start-up period, the influent flowrate was maintained at 1.6 mL/min (resulting in an HRT of 5.8 h), and the H_2_ and CO_2_ pressure was kept at 0.04 and 0.012 MPa, respectively. After 30 days of operation, the influent flowrate was changed to 2.0 mL/min (corresponding to an HRT of 4.7 h) until the biofilm was well formed, and the performance of the reactor reached a steady state with stable NO_3_^−^ removal.

### 2.2. Short- and Long-Term Experiments and Sample Analysis

On days 31–40 of stage 1, short-term experiments were performed to assess the effects of H_2_ pressure (0.01–0.08 MPa), influent NO_3_^−^ (1–20 mg N/L), and CO_2_ pressure (0.004–0.036 MPa) on the NO_3_^−^ and BrO_3_^−^ removal of the H_2_-MBfR. The detailed information concerning the operational conditions of short-term experiments can be found in Table S1. For each experiment, one system operating parameter was changed, while keeping the others fixed. Once the experiment condition was changed, aqueous samples were collected after three HRT operations of the system, in order to allow the stabilization of effluent contaminant concentrations [18]. Following the short-term experiments, long-term experiments (days 41–140) were carried out to investigate how the oxyanion removal performance of the system responded to the changing BrO_3_^−^ loadings. As summarized in Table S2, in long-term experiments, the influent concentrations of BrO_3_^−^ were set at 0.1, 0.5, and 1.0 mg/L in stage 3, stage 4, and stage 5, respectively, and the H_2_ and CO_2_ pressure were maintained at 0.04 and 0.012 MPa, respectively.

The collected aqueous samples were filtered immediately through a 0.22 µm pore size polycarbonate membrane filter (Anpel Co., Ltd., Shanghai, China) and stocked in a refrigerator (4 °C) until analyzed. The concentrations of NO_3_^−^, NO_2_^−^, and BrO_3_^−^ were determined by ion chromatography (ICS-1000, Dionex, Sunnyvale, USA) equipped with an AS-19 column (4 × 250 mm, Dionex, Sunnyvale, USA). The dissolved H_2_ was determined using a H_2_ microsensor (H_2_10, Unisense A/S Corp., Aarhus, Denmark). The pH value was measured using a pH meter (pHS-3C model, Leici, Shanghai, China). The contaminant removal flux was calculated using Equation (1):

(1) $$J=\frac{Q}{A}\left(S_{i}-S_{e}\right),$$

where *J* is the NO_3_^−^ and BrO_3_^−^ removal flux in units of g N/(m^2^·day) and g BrO_3_^−^/(m^2^·day), respectively, *Q* signifies the influent flowrate (m^3^/day), *A* refers to the total membrane surface area (m^2^), and *S*_i_ and *S*_e_ denote the influent concentration of NO_3_^−^ or BrO_3_^−^ and the effluent concentration of NO_3_^−^ or BrO_3_^−^, respectively.

### 2.3. Model Development and Evaluation

#### 2.3.1. Model Framework Development

**Figure 2:**
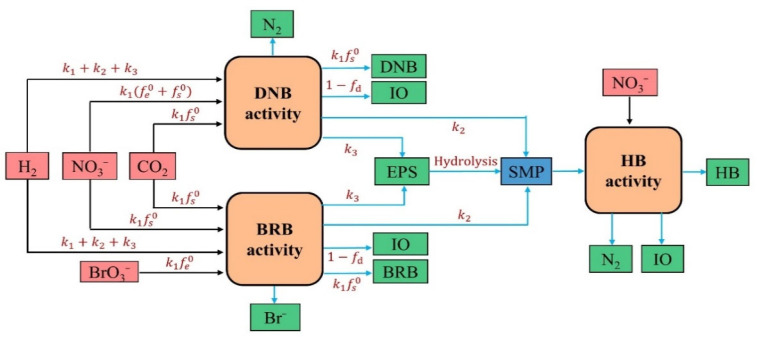
Schematic of the interactions between model components involved in the biochemical processes of the biofilm.

Unlike the classical biofilm models established by Tang et al. [14,16], the framework of the developed mathematical model involves the biochemical process of hydrogenotrophic BrO_3_^−^ reduction, and it considers the effects on the metabolic process kinetics of BRB and DNB in the case of CO_2_ addition (as carbon source and pH regulator). Figure 2 shows the schematic of the involved biochemical processes and the correlated interactions between the model components in the biofilm. The model components include five solid components (i.e., BRB, DNB, heterotrophic bacteria (HB), extracellular polymeric substances (EPS), and inert organics (IO)) and five dissolved components (i.e., H_2_, CO_2_, NO_3_^−^, BrO_3_^−^, and soluble microbial products (SMP)). H_2_ is exploited by BRB and DNB as the electron donors, BrO_3_^−^ and NO_3_^−^ as the electron acceptors, and CO_2_ as the carbon source. In the conversion processes of BrO_3_^−^ and NO_3_^−^ to Br^−^ and N_2_, BRB and DNB obtain energy to sustain their growth and metabolism. H_2_ was the sole energy source of the hydrogenotrophic bacteria, since no organic carbon was introduced to the influent; thus, BRB and DNB could merely abstract electrons from H_2_ to drive the BrO_3_^−^ and NO_3_^−^ reduction. HB grows either on the SMP directly produced by BRB and DNB or indirectly on the hydrolyzed EPS (i.e., SMP) originated from BRB and DNB. The decay of BRB, DNB, and HB generates nonbiodegradable IO. The electrons provided from H_2_ can be classified into three portions during the BRB and DNB metabolic processes: bacterial cell synthesis (*k*_1_), SMP formation (*k*_2_), and EPS formation (*k*_3_); thus, *k*_1_ + *k*_2_ + *k*_3_ = 1. The electrons for cell synthesis are subdivided into the fraction for energy-providing reaction (*f*_e_^0^) and the other for synthesis reaction of true yield of biomass (*f*_s_^0^); therefore, *f*_e_^0^ + *f*_s_^0^ = 1. Here, *f*_d_ represents the fraction of biodegradable biomass; hence, 1 − *f*_d_ refers to the remaining nonbiodegradable biomass.

#### 2.3.2. Model Solution and Calibration

The numerical solution of the model was implemented on software AQUASIM 2.1g, by inputting the process matrix (Table 1), stoichiometric and kinetic parameters (Table S3), process kinetic rate equations (Table 2), and experiment operational parameters of the H_2_-MBfR (Table S4). In particular, the calculation procedure for stoichiometric coefficients of model components (shown in Table 1) in the BRB metabolic process is exhibited in Section S1. Table S3 merely lists the calculated and recalibrated stoichiometric and kinetic parameters of the BRB metabolic process in this work, while other pertinent parameters can be found in Table S5. Bulk liquid pH is known to be a key factor correlated to the activities of the hydrogenotrophic microorganisms [13,19,20]. In order to quantitatively assess how bulk liquid pH affects the microbial metabolic activities, the inhibition factor of bulk liquid pH (*f*_pH_) was introduced to the kinetic rate expressions of the model framework, as shown in Table 2. According to the normalized Michaelis pH function [21], *f*_pH_ can be computed using Equation (2).

(2) $$f_{pH}=\frac{1+2\cdot 10^{0.5\left(pK_{l}-pK_{h}\right)}}{1+10^{\left(pH-pK_{h}\right)}+10^{\left(pK_{l}-pH\right)}},$$

where pH values are the experimental measurements in this study; *pK*_h_ and *pK*_l_ denote the upper and lower pH values at which the metabolic rates are equal to 50% of the maximum rate at the optimum pH, respectively, and their values of 9.04 and 6.27 were adopted from previous studies [21,22,23].

Disparate reactor configurations, operating conditions, and HFM patterns commonly result in the varying compositions and metabolic kinetics of biofilms [24]. For the aim of matching the specific simulation scenarios, several parameters, i.e., H_2_ transfer coefficient of HFM (*K*_m_), maximum specific growth rate of DNB and BRB (*µ*_DNB_ and *µ*_BRB_), and half-maximum-rate concentration of BrO_3_^−^ for BRB (*K*_BrO3_), were recalibrated by fitting the measured effluent NO_3_^−^ and BrO_3_^−^ concentrations of the long-term operated system to the modeled results using the AQUASIM built-in iterative algorithms (Equation (3)). By minimizing the square error sums between the model predictions and experimental measurements, the selected parameters were calibrated to be the best-fit values.

(3) $$\chi^{2}\left(p\right)={\sum_{i=1}^{n}\left(\frac{y_{meas,i}-y_{i}\left(p\right)}{\sigma_{meas,i}}\right)}^{2},$$

where *y*_meas,i_ and *y*_i_(*p*) represent the experimental results and model predictions at time i, and *σ*_meas;i_ and n signify the standard deviation and number of data points, respectively.

#### 2.3.3. Model Validation and Evaluation

**Figure 3:**
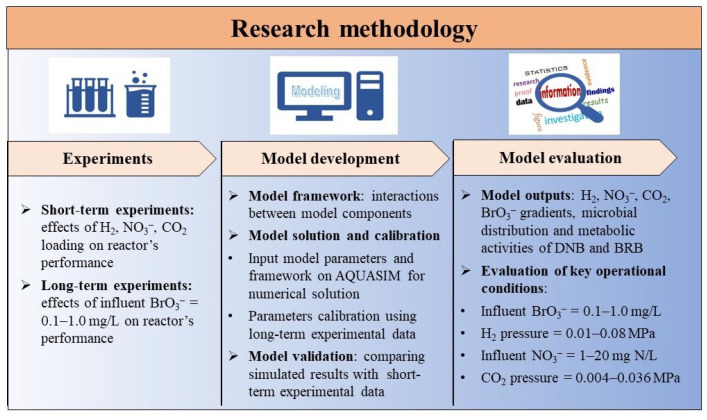
Schematic of the research methodology.

The calibrated model was further validated by comparing the simulated results with experimentally measured NO_3_^−^ and BrO_3_^−^ removal fluxes in the short-term experiments. The validated model was then used to simulate the profiles of substrates, microbial distribution, and metabolic activities in the biofilm of the H_2_-MBfR operated in a series of system conditions, including influent BrO_3_^−^ concentrations (0.1–1.0 mg/L), H_2_ pressure (0.01–0.08 MPa), influent NO_3_^−^ concentration (1–20 mg N/L), and CO_2_ pressure (0.004–0.036 MPa), in order to investigate the evolution laws of biofilm microenvironment, structure, and activity in the H_2_-MBfR for NO_3_^−^ and BrO_3_^−^ removal, in the case of changing system conditions. A schematic of the research methodology is shown in Figure 3.

## 3. Results and Discussion

### 3.1. Long-Term H_2_-MBfR Performance and Model Calibration

**Figure 4:**
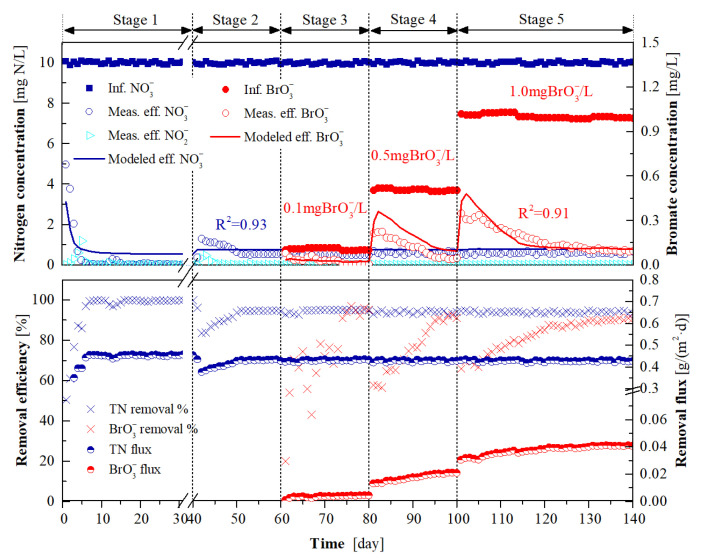
Long-term performance of NO_3_^−^ and BrO_3_^−^ reduction in the H_2_-MBfR. The abbreviations “Inf.”, “Eff.”, and “Meas.” denote influent, effluent, and measured, respectively.

**Figure 5:**
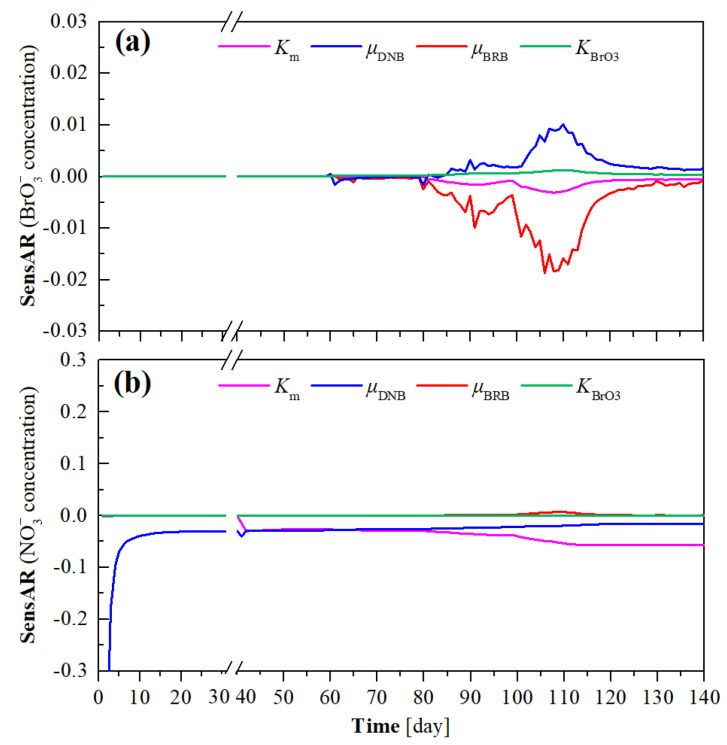
Sensitivities of the effluent BrO_3_^−^ (**a**) and NO_3_^−^ (**b**) concentrations to the selected parameters *K*_m_, *µ*_DNB_, *µ*_BRB_, and *K*_BrO3_.

The long-term experimental data obtained during the 140 days of operation of H_2_-MBfR were used for the recalibration of the selected parameters by fitting the effluent NO_3_^−^ and BrO_3_^−^ concentrations to those of simulated results. As shown in Figure 4, the measured data and simulated results were finely matched with the corresponding coefficients of determination (*R^2^*) up to 0.93 and 0.91 for the effluent NO_3_^−^ and BrO_3_^−^ concentrations, respectively. The analysis results concerning the sensitivity of the selected parameters to the effluent BrO_3_^−^ and NO_3_^−^ concentrations in the long-term operated H_2_-MBfR are shown in Figure 5. It can be found that the effluent BrO_3_^−^ concentration of the H_2_-MBfR was most sensitive to *µ*_BRB_ and relatively sensitive to *µ*_DNB_ and *K*_m_ (Figure 5a), while the effluent NO_3_^−^ concentration was sensitive to *K*_m_ and *µ*_DNB_ (Figure 5b). These results are in line with the findings of previous studies, i.e., the maximum specific growth rate of microorganisms and the H_2_ transfer coefficient of HFM were sensitive to the H_2_-MBfR performance [9,24,25].The best-fit values of *K*_m_, *µ*_BRB_, *µ*_DNB_, and *K*_BrO3_ were estimated to be 0.189 m/day, 0.85 day^−1^, 0.57 day^−1^, and 0.014 mg/L, respectively.

It can be also seen from Figure 4 that, after 7 days of start-up at stage 1, the denitrification flux of the H_2_-MBfR stabilized at around 0.45 g N/(m^2^·day), corresponding to a NO_3_^−^ removal efficiency of over 99%. The slightly decreased denitrification flux of the system at stage 2 can be ascribed to the augment in the influent flowrate from 1.6 mL/min at stage 1 to 2.0 mL/min at this stage. The introduction of 0.1–1.0 mg/L BrO_3_^−^ into the influent at stages 3–5 did not further decrease the denitrification performance of the system, given the identical average denitrification flux (0.42 g N/(m^2^·day)) at days 50–60 of stage 2 (without BrO_3_^−^ addition) and stages 3–5. This observation is in agreement with the results of previous studies that a BrO_3_^−^ concentration below 50 mg/L had no inhibitory effect on denitrification [15,26]. Empirical studies suggest that it usually takes 11–40 days to enable the H_2_-MBfRs to reach steady-state characteristics following the adjustment of operation conditions such as substrate loadings and HRT [17,27]. Similarly, our results indicate that, in the case of BrO_3_^−^ addition, it took 15–25 days at stages 3–5 to enable the BrO_3_^−^ removal of the H_2_-MBfR to reach a steady state. The stabilized BrO_3_^−^ removal flux was increased from 0.0044 g/(m^2^·day) at stage 3 to 0.041 g/(m^2^·day) at stage 5. This is presumably attributed to the augment in the abundance and/or reduction rate of BRB with increasing influent BrO_3_^−^ concentration. Furthermore, Downing and Nerenberg [15] found that a near 11-fold increase in the BrO_3_^−^ removal flux of H_2_-MBfR was achieved when the influent BrO_3_^−^ concentration was increased from 0.1 to 10 mg/L.

### 3.2. Short-Term H_2_-MBfR Performance and Model Validation

**Figure 6:**
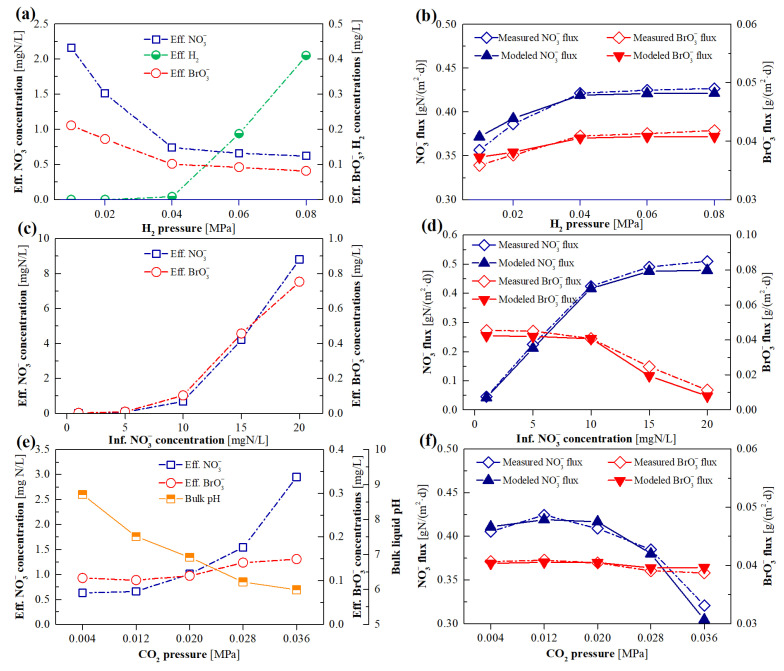
The system performance of the H_2_-MBfR, as well as the compassion of the measured and simulated removal fluxes of NO_3_^−^ and BrO_3_^−^, in the case of changing H_2_ pressure (**a**,**b**), influent NO_3_^−^ concentration (**c**,**d**), and CO_2_ pressure (**e**,**f**).

Short-term experiments were performed to evaluate the effects of the key influencing factors, i.e., H_2_ pressure, influent NO_3_^−^ concentration, and CO_2_ pressure, on the NO_3_^−^ and BrO_3_^−^ reduction of the H_2_-MBfR. As shown in Figure 6a,b the determined BrO_3_^−^ and NO_3_^−^ removal fluxes were increased from 0.036 and 0.36 to 0.041 g/(m^2^·day) and 0.42 g N/(m^2^·day), respectively, as the H_2_ pressure was increased from 0.01 to 0.04 MPa. This is likely owing to the increased availability of electron donor, which enhanced the activities of BRB and DNB. A higher H_2_ pressure (0.06 and 0.08 MPa) did not ameliorate the BrO_3_^−^ and NO_3_^−^ removal, but led to the consumption of the remaining H_2_ in the effluent. Figure 6c,d delineate that, although the BrO_3_^−^ and NO_3_^−^ were all accumulated in the effluent, the NO_3_^−^ removal flux was increased from 0.42 to 0.51 g N/(m^2^·day), accompanied by the significant decrease in BrO_3_^−^ removal flux from 0.041 to 0.011 g/(m^2^·day), when the influent NO_3_^−^ concentrations were augmented from 10 to 20 mg N/L. In accordance with this, Zhong et al. [6] observed that an influent NO_3_^−^ concentration of 11.3 mg N/L was able to inhibit the BrO_3_^−^ reduction in the H_2_-MBfR, which gave rise to the advantage of DNB over BRB when competing for electron donors. CO_2_ is arguably one of the most effective pH regulators in H_2_-MBfRs, and it can serve as the supplemental inorganic carbon source for hydrogenotrophic bacteria [13,28]. As depicted in Figure 6e,f, the shift of CO_2_ pressure from 0.012 to 0.036 MPa led to an apparent decline in the NO_3_^−^ removal flux from 0.42 g N/(m^2^·day) at 0.012 MPa to 0.32 g N/(m^2^·day) at 0.036 MPa, but had a negligible influence on BrO_3_^−^ removal flux. A plausible explanation is that, as the CO_2_ pressure was in the range of 0.020–0.036 MPa, the resultant bulk liquid pH of 6.0–6.9 was obviously lower than the reported favorable pH range (7.0–9.0) of DNB, according to existing studies [19,20], and some specific BRB was possibly able to endure more acidic conditions. Additionally, the measured removal fluxes of BrO_3_^−^ and NO_3_^−^ in the short-term experiments were employed to further validate the calibrated model. It can be observed from Figure 6b–f that, in all cases, the modeled results were quite approximate to the experimental data, indicating the good accuracy and reliability of the developed model for prediction of the simultaneous BrO_3_^−^ and NO_3_^−^ reduction processes in the H_2_-MBfR.

### 3.3. Model Evaluation of the Effects of BrO_3_^−^ Loading

**Figure 7:**
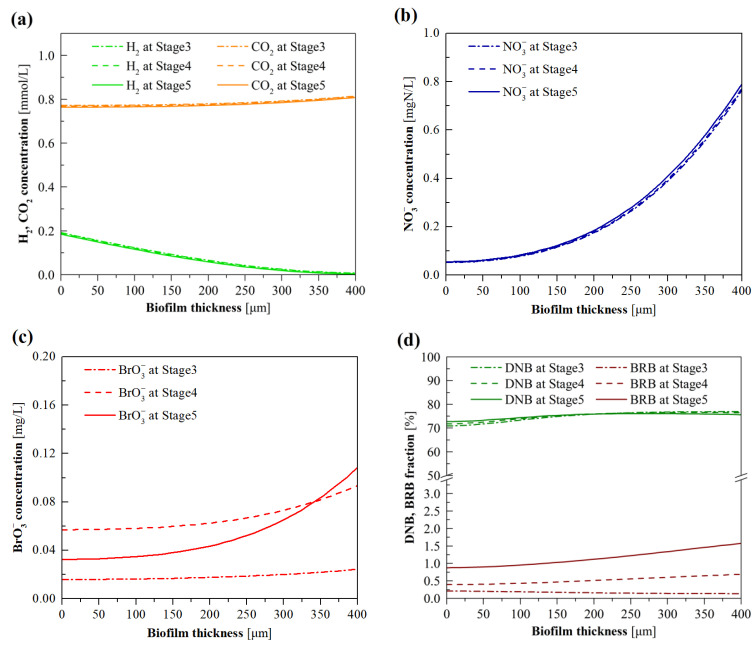

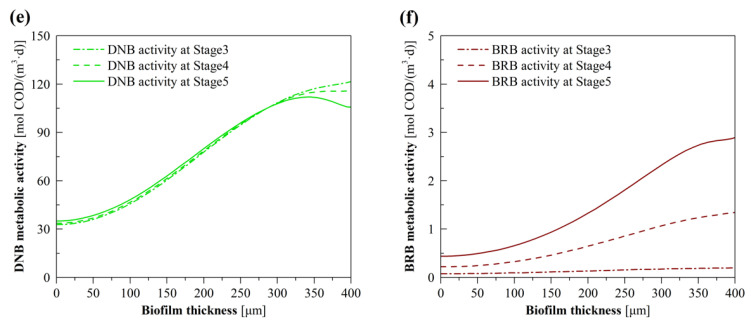
Model-simulated profiles for (**a**) H_2_ and CO_2_, (**b**) NO_3_^−^, (**c**) BrO_3_^−^ concentrations, and (**d**) DNB and BRB fractions, as well as (**e**) DNB and (**f**) BRB metabolic activities, in the biofilm of H_2_-MBfR as a function of influent BrO_3_^−^ concentration ranging from 0.1 to 1.0 mg/L.

Figure 7 shows the simulated distribution of substrates, DNB and BRB fractions, as well as their metabolic activities, in the steady-state biofilms at stages 3–5 using the validated model. As shown in Figure 7a–c, in line with the counter-diffusional characteristics of dissolved substrate in H_2_-MBfR biofilm [29], the H_2_ concentration gradually decreased from HFM surface (i.e., biofilm thickness of zero point) toward to the bulk liquid side, while the other substrates (including CO_2_, NO_3_^−^, and BrO_3_^−^) originated from bulk liquid presented the contrary tendency. H_2_ and CO_2_ concentrations across the biofilm exhibited negligible difference when the influent concentration varied in the range of 0.1–1.0 mg/L (Figure 7a), attributed to the limited electron donor and carbon source consumption by BRB at low BrO_3_^−^ loadings. It can also be seen from Figure 7a that CO_2_ was not a limiting factor as a carbon source for BRB and DNB anabolism, since its concentrations within the whole biofilm were much higher than the half-maximum-rate concentration (0.004 mg/L) adopted in the model. Moreover, the resultant bulk liquid pH 7.5 at the CO_2_ pressure of 0.012 MPa would not adversely affect the metabolic processes of BRB and DNB, since the maximum NO_3_^−^ and BrO_3_^−^ removal fluxes were attained at this CO_2_ pressure, as shown in Figure 6f. In line with this, the optimal pH reported for hydrogenotrophic denitrification and bromate reduction has been documented at neutral or weakly alkaline values [13,15].

Figure 7d plots the simulated profiles of DNB and BRB fractions in the biofilm. DNB dominated in the biofilm compared to BRB at all stages, indicating that DNB outcompeted DNB for space, likely associated with the much higher concentration of NO_3_^−^ in the biofilm than BrO_3_^−^. In particular, in response to the increase of the influent BrO_3_^−^ concentration from stage 3 to stage 5, the BRB fraction (proportion) obviously increased in the biofilm. Figure 7e shows the DNB activity in the biofilm as a function of influent BrO_3_^−^ concentration. The declined DNB activity in the biofilm range of 0–210 μm was mainly due to the inhibition of electron acceptors for DNB metabolism, given that the NO_3_^−^ concentrations within this range were below the half-maximum-rate concentration of NO_3_^−^ for DNB (0.2 mg N/L) [14]. The observed decrease in DNB activity from stage 3 to stage 5 at the outer layer biofilm was the result of the lower H_2_ concentrations. As shown in Figure 7f, the BRB activity in the biofilm increased from stage 3 to stage 5, owing to the augmented BRB fraction and BrO_3_^−^ gradients in the biofilm, which facilitated the metabolism of BRB. In addition, the increased BRB fraction and activity, as exhibited in Figure 7d,f, helps explain the augmented BrO_3_^−^ removal flux from stage 3 to stage 5 (Figure 4).

### 3.4. Model Evaluation of the Effects of H_2_ Pressure

**Figure 8:**
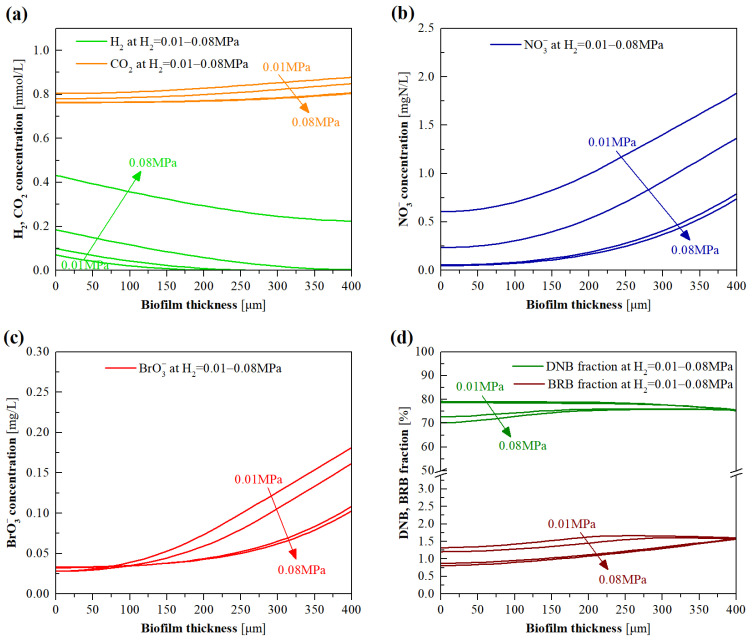

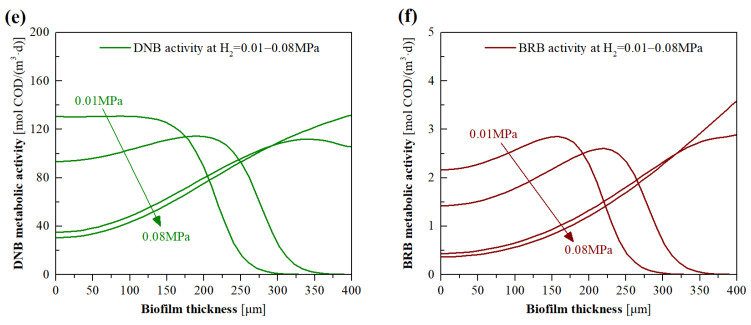
Model simulated profiles for (**a**) H_2_ and CO_2_, (**b**) NO_3_^−^, (**c**) BrO_3_^−^ concentrations, and (**d**) DNB and BRB fractions, as well as (**e**) DNB and (**f**) BRB metabolic activities, in the biofilm of H_2_-MBfR as a function of H_2_ pressure ranging from 0.01 to 0.08 MPa.

Empirical studies [13,20] have collectively shown that excessive H_2_ supply pressure results in the H_2_ off-gassing problem, while an insufficient supply leads to unsatisfactory pollutant removal due to the electron donor scarcity in biofilms. Figure 8a plots the simulated H_2_ and CO_2_ concentration profiles in the biofilm at diverse H_2_ pressure (0.01–0.08 MPa). The H_2_ concentration in the biofilm increased with the increase in H_2_ pressure. At the H_2_ pressure of 0.01 MPa, more than half of the outer biofilm region (biofilm thickness >200 μm) could not deliver sufficient H_2_. H_2_ constitutes a limiting factor for microbial metabolic processes when its content is below the half-maximum-rate concentration. When H_2_ pressure was increased to 0.04 MPa, the H_2_ contents across the biofilm were all higher than the half-maximum-rate concentrations of H_2_ for DNB and BRB metabolism (0.002 mg/L) [14]; thus, it can be inferred that the H_2_ concentration within biofilm was not a decisive factor limiting NO_3_^−^ and BrO_3_^−^ removal at relatively high H_2_ pressure. Note that, at this H_2_ pressure, the simulated H_2_ concentration in the bulk liquid was 0.0074 mg/L, which is quite close to the measured result (0.008 mg/L) during the short-term experiments (Figure 6a) and is also comparable to the recommended aquatic H_2_ concentration (0.009 mg/L) in H_2_-MBfR [18]. Upon further increasing the H_2_ pressure, the H_2_ off-gassing phenomenon occurred. It can also be seen from Figure 8a that the CO_2_ concentration gradients in the biofilm hardly changed when the H_2_ pressure was higher than 0.04 MPa. This implies that the excessive H_2_ supply would not accelerate the carbon source utilization of BRB and DNB.

As depicted in Figure 8b,c, with the increase in H_2_ pressure from 0.01 to 0.04 MPa, the NO_3_^−^ and BrO_3_^−^ concentrations in the biofilm were found to markedly decline. This can be explained by the increased H_2_ availability for DNB and BRB compared to the biofilm exterior, which allowed a greater reduction in NO_3_^−^ and BrO_3_^−^ in the exterior rather than the interior of biofilm. However, NO_3_^−^ and BrO_3_^−^ concentration gradients hardly varied once the H_2_ pressure exceeded 0.04 MPa, which is in accordance with the experimental findings of the short-term experiments that the NO_3_^−^ and BrO_3_^−^ removal marginally changed at the excessive H_2_ pressure (shown in Figure 6b). According to the simulated BrO_3_^−^ concentration profiles, BrO_3_^−^ concentrations in the biofilm were all greater than its calibrated half-maximum-rate concentration (0.014 mg/L) at the simulated H_2_ pressure range, indicating that BrO_3_^−^ loading was also not a limiting factor to BRB activity. The increased H_2_ availability in the outer layer of the biofilm resulted in the extension of the active region of DNB and BRB from the biofilm interior to the whole biofilm, as revealed in Figure 8e,f. In particular, the decreased activities of DNB and BRB in the inner layer of biofilm with increasing H_2_ pressure were mainly the consequence of the concurrently decreased concentrations of NO_3_^−^ and BrO_3_^−^ (Figure 8b,c) and the fractions of DNB and BRB (Figure 8d). In the case of low H_2_ pressure (0.01 and 0.02 MPa), the biofilm thicknesses of 200 and 260 μm were the locations where the H_2_ concentrations were all below the half-maximum-rate concentrations of DNB and BRB (Figure 8a); beyond these thicknesses, the metabolic activities of DNB and BRB began to dramatically decrease, as delineated in Figure 8e,f.

### 3.5. Model Evaluation of the Effects of NO_3_^−^ Loading

**Figure 9:**
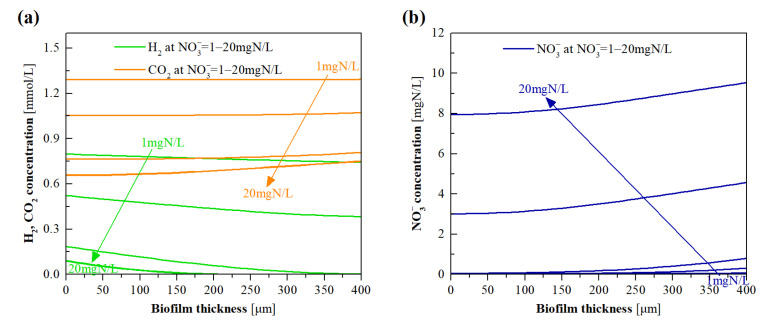

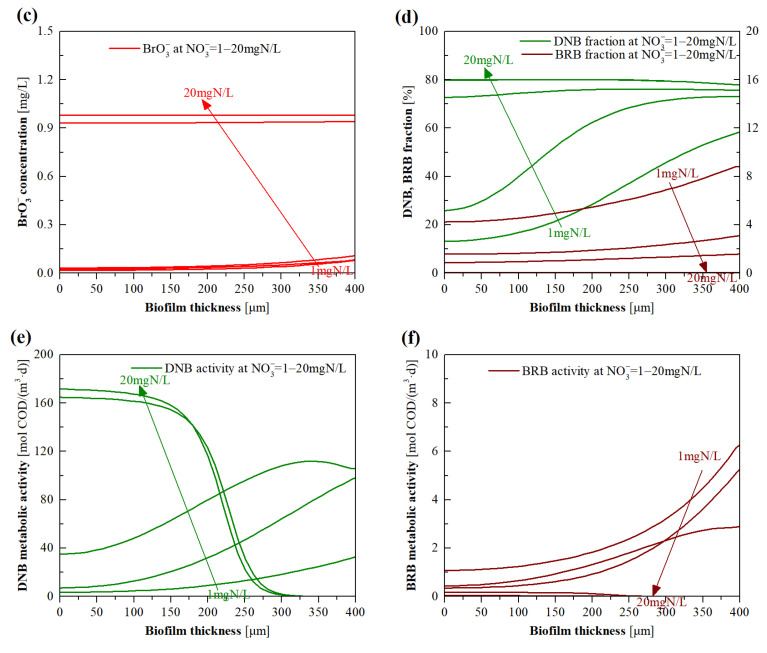
Model simulated profiles for (**a**) H_2_ and CO_2_, (**b**) NO_3_^−^, (**c**) BrO_3_^−^ concentrations, and (**d**) DNB and BRB fractions, as well as (**e**) DNB and (**f**) BRB metabolic activities, in the biofilm of H_2_-MBfR as a function of influent NO_3_^−^ concentration ranging from 1 to 20 mg N/L.

The experimental results, as shown in Figure 6c, corroborate that the NO_3_^−^ loading significantly impacted the BrO_3_^−^ removal of the H_2_-MBfR. To understand the underlying mechanism, we employed the validated model to simulate the substrate profiles and microbial activities in the biofilm at the influent NO_3_^−^ concentrations of 1–20 mg N/L. As shown in Figure 9a, the H_2_ and CO_2_ concentration gradients in the biofilm sharply decreased with increasing influent NO_3_^−^ concentration, implying that high NO_3_^−^ loadings might signally enhance the activity of DNB, leading to greater consumption of these two substrates. In particular, H_2_ off-gassing was found at the influent NO_3_^−^ concentration lower than 10 mg N/L, owing to the lack of an electron acceptor for H_2_ consumption. Once the influent NO_3_^−^ concentration was greater than 10 mg N/L, NO_3_^−^ was found to accumulate in the biofilm with high concentrations (Figure 9b), presumably due to the limitation of overall DNB population, while over 90% of the inputted BrO_3_^−^ could not be consumed by BRB in the biofilm (Figure 9c), suggesting the severe inhibition of BRB activity by high NO_3_^−^ loadings. As exhibited in Figure 9d, when the influent NO_3_^−^ concentration was increased from 1 to 20 mg N/L, the BRB factions across the biofilm decreased from beyond 20% to approximately 1%, which was accompanied by the surge of the DNB fraction across the biofilm, suggesting the competitive advantage of DNB over BRB in the case of the high NO_3_^−^ loadings.

Figure 9e,f show the simulated DNB and BRB metabolic activities in the biofilm of the system operated under various influent NO_3_^−^ concentrations. As the influent NO_3_^−^ concentration was less than 10 mg N/L, the high DNB and BRB activities appeared at the outer layer of the biofilm, and a closer distance from the HFM side led to lower activities of DNB and BRB. This trend coincides with that of NO_3_^−^ and BrO_3_^−^ concentration gradients in the biofilm, as shown in Figure 9b,c. When the influent NO_3_^−^ concentration was higher than 10 mg N/L, the DNB activities sharply dropped at the biofilm thickness of beyond 210 μm. This is because the H_2_ concentrations in the locations beyond this thickness were lower than the half-maximum-rate concentration of DNB (0.002 mg/L). In addition, the BRB activities were strongly inhibited at the higher influent NO_3_^−^ concentration (especially 20 mg N/L), indicating that BRB was overwhelmed by DNB for electron donor competition, when NO_3_^−^ concentration in the influent was much higher than that of BrO_3_^−^. This is consistent with the previous findings that an excessive influent NO_3_^−^ concentration could inhibit BrO_3_^−^ reduction [6,30].

### 3.6. Model Evaluation of the Effects of CO_2_ Pressure

**Figure 10:**
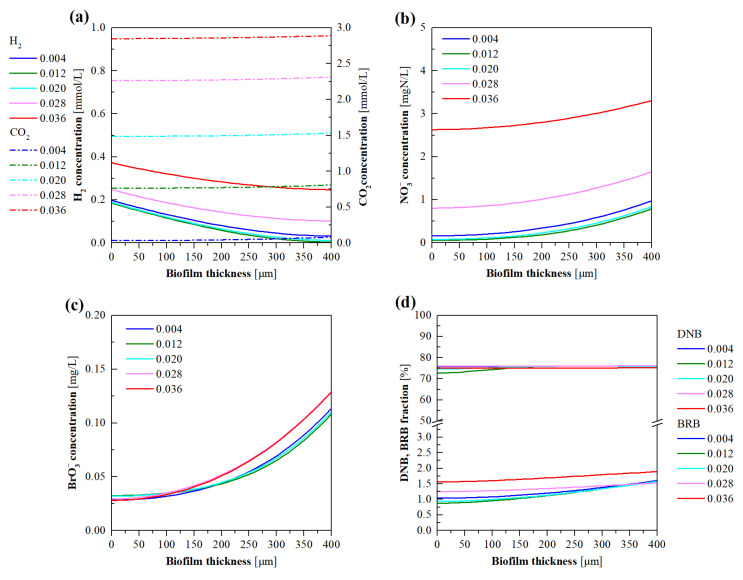

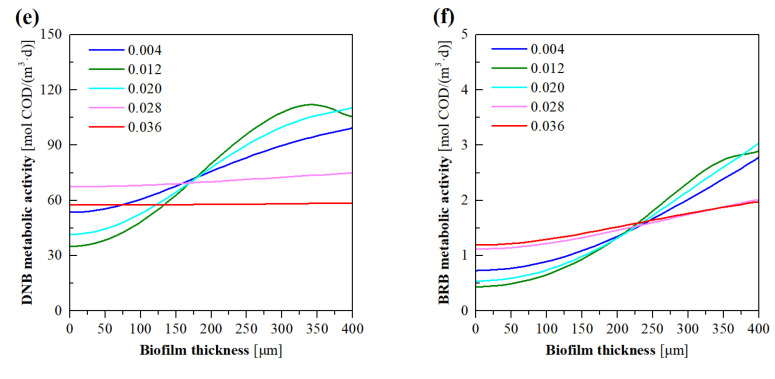
Model simulated profiles for (**a**) H_2_ and CO_2_, (**b**) NO_3_^−^, (**c**) BrO_3_^−^ concentrations, and (**d**) DNB and BRB fractions, as well as (**e**) DNB and (**f**) BRB metabolic activities, in the biofilm of H_2_-MBfR as a function of CO_2_ pressure ranging from 0.004 to 0.036 MPa.

CO_2_ has dual functions, i.e., as a carbon source to support microorganism growth and for pH control to regulate the microbial activities in H_2_-MBfRs [19,31]. Simulations were, therefore, conducted to investigate the effects of CO_2_ addition on the evolution of stratification characteristics of the biofilm at the supply pressure of 0.004–0.036 MPa. As shown in Figure 10a–c, the increase in CO_2_ pressure from 0.004 to 0.02 MPa led to insignificant changes in the H_2_, NO_3_^−^, and BrO_3_^−^ concentration gradients of the biofilm. Once the CO_2_ pressure was higher than 0.02 MPa, the accumulation of H_2_ and NO_3_^−^ was increased in the biofilm. The BrO_3_^−^ concentrations in the biofilm were slightly increased at the CO_2_ pressure of 0.036 MPa. This implies that DNB was more sensitive to the acidic conditions (pH 6.0–6.2 at CO_2_ pressure 0.028–0.036 MPa) than BRB.

As depicted in Figure 10d, as the CO_2_ pressure was increased from 0.004 to 0.036 MPa, no apparent difference was found in the DNB fractions, while a slight increase in the BRB fractions was observed. It can be seen from Figure 10e,f that, when the CO_2_ pressure was less than 0.02 MPa, the DNB and BRB activities in the biofilm all gradually augmented with the increase in distance from the HFM side. This can be attributed to the relatively higher NO_3_^−^ and BrO_3_^−^ concentrations in the biofilm exterior. It is interesting to note that, compared to the activities of DNB and BRB at the CO_2_ pressure below 0.02 MPa, a CO_2_ pressure beyond this value gave rise to decreased and increased microbial activities in the exterior and interior of the biofilm, respectively. This can be explained by the fact that DNB and BRB in the biofilm exterior were in the vicinity of the bulk liquid, and the acidification of bulk liquid severely inhibited their metabolic activities; subsequently, the declined activities of microorganisms in the biofilm exterior led to more diffusion of NO_3_^−^ and BrO_3_^−^ toward the HFM side (Figure 10b,c), which consequently led to increased activities of DNB and BRB in the biofilm interior. When the CO_2_ pressure was increased from 0.012 to 0.036 MPa, DNB activity in the majority of the biofilm significantly declined, while the decreased BRB activity in the biofilm exterior was accompanied by an increase in BRB activity in the biofilm interior (Figure 10e,f). This also indicates that BRB was more tolerant of the acidic aquatic environment than DNB.

## 4. Conclusions

An expanded multispecies model was developed to provide key information regarding the microbial BrO_3_^−^ and NO_3_^−^ reduction processes in the H_2_-MBfR in diverse operating conditions. The selected model parameters, *K*_m_, *µ*_BRB_, *µ*_DNB_, and *K*_BrO3_, were calibrated to the best-fit values of 0.189 m/day, 0.85 day^−1^, 0.57 day^−1^, and 0.014 mg/L, respectively. The good agreement between experimental measurements and modeled results indicates the accuracy and reliability of the calibrated model. The evolution of the substrate gradients, as well as microbial fraction and activity, was driven by the changing system operating parameters involving H_2_ pressure, BrO_3_^−^ and NO_3_^−^ loading, and CO_2_ pressure. Increasing H_2_ pressure led to more efficient BrO_3_^−^ and NO_3_^−^ reduction in the biofilm, but an exorbitant pressure (beyond 0.04 MPa) gave rise to off-gassing of H_2_. The augment in BrO_3_^−^ loading had no significant influence on NO_3_^−^ reduction, while an influent NO_3_^−^ concentration higher than 10 mg N/L resulted in the apparent inhibition of BrO_3_^−^ reduction. A CO_2_ pressure over 0.02 MPa had a distinct negative influence on NO_3_^−^ reduction, but minorly impacted BrO_3_^−^ reduction. The simulation results of the developed model offer important mechanistic insights into the BrO_3_^−^ and NO_3_^−^ reduction processes of H_2_-MBfR.

## Figures and Tables

**Table 1:** Process matrix for the developed model of components.

Component (i) Process (j)	S_H2_	S_NO3_	S_BrO3_	S_SMP_	S_CO2_	X_DNB_	X_BRB_	X_HB_	X_IO_	X_EPS_
DNB growth	$-\frac{14}{5f_{s,\mathrm{DNB}}^{0}}$	$-k_{1}\left(\frac{28f_{e,\mathrm{DNB}}^{0}}{25f_{s,\mathrm{DNB}}^{0}}+\frac{1}{5}\right)$		*k*_2_	−*k*_1_	*k*_1_				*k*_3_
BRB growth	$-\frac{14}{5f_{s,\mathrm{BRB}}^{0}}$	$-\frac{1}{5}k_{1}$	$-k_{1}\frac{14f_{e,\mathrm{DNB}}^{0}}{15f_{s,\mathrm{DNB}}^{0}}$	*k*_2_	−*k*_1_		*k*_1_			*k*_3_
HB growth		$-\frac{1-Y_{\mathrm{HB}}}{1.25Y_{\mathrm{HB}}}$		$-\frac{1}{Y_{\mathrm{HB}}}$				1		
DNB decay						−1			1−*f*_d_	
BRB decay							−1		1−*f*_d_	
HB Decay								−1	1−*f*_d_	
Hydrolysis				1						−1
	H_2_ (mol/m^3^)	NO_3_ (mol/m^3^)	BrO_3_ (mol/m^3^)	SMP (mol/m^3^)	CO_2_ (mol/m^3^)	DNB (mol/m^3^)	BRB (mol/m^3^)	HB (mol/m^3^)	IO (mol/m^3^)	EPS (mol/m^3^)

**Table 2:** Process kinetic rate equations for the developed model.

Process (j)	Kinetic Rate Expressions
DNB growth	$\mu_{\mathrm{DNB}}\frac{S_{H2}}{S_{H2}+K_{H2}^{\mathrm{DNB}}}\frac{S_{\mathrm{NO}3}}{S_{\mathrm{NO}3}+K_{\mathrm{NO}3}^{\mathrm{DNB}}}\frac{S_{\mathrm{CO}2}}{S_{\mathrm{CO}2}+K_{\mathrm{CO}2}^{\mathrm{DNB}}}X_{\mathrm{DNB}}f_{\mathrm{pH}}^{}$
BRB growth	$\mu_{\mathrm{BRB}}\frac{S_{H2}}{S_{H2}+K_{H2}^{\mathrm{BRB}}}\frac{S_{\mathrm{NO}3}}{S_{\mathrm{NO}3}+K_{\mathrm{NO}3}^{\mathrm{BRB}}}\frac{S_{\mathrm{BrO}3}}{S_{\mathrm{BrO}3}+K_{\mathrm{BrO}3}^{\mathrm{BRB}}}\frac{S_{\mathrm{CO}2}}{S_{\mathrm{CO}2}+K_{\mathrm{CO}2}^{\mathrm{BRB}}}X_{\mathrm{BRB}}f_{\mathrm{pH}}^{}$
HB growth	$\mu_{\mathrm{HB}}\frac{S_{\mathrm{NO}3}}{S_{\mathrm{NO}3}+K_{\mathrm{NO}3}^{\mathrm{HB}}}\frac{S_{\mathrm{SMP}}}{S_{\mathrm{SMP}}+K_{\mathrm{SMP}}^{\mathrm{HB}}}X_{\mathrm{HB}}f_{\mathrm{pH}}^{}$
DNB decay	$b_{\mathrm{DNB}}X_{\mathrm{DNB}}$
BRB decay	$b_{\mathrm{BRB}}X_{\mathrm{BRB}}$
HB Decay	$b_{\mathrm{HB}}X_{\mathrm{HB}}$
Hydrolysis	$k_{\mathrm{hyd}}X_{\mathrm{EPS}}$

## Data Availability

Not applicable.

## Supplementary Materials

The following supporting information can be downloaded at https://www.mdpi.com/article/10.3390/membranes12080774/s1: Table S1. Short-term operational conditions of influencing factor experiment of the H_2_-MBfR; Table S2. Start-up and long-term operational conditions of the H_2_-MBfR; Table S3. Stoichiometric and kinetic parameters of BRB related metabolic process for the developed model; Table S4. Experiments operational parameters for the model development; Table S5. Other pertinent input parameters for model development; Section S1. Calculation of the stoichiometric coefficients for BRB metabolic process. Refs. [32,33] are cited on Supplementary Materials.
