# Supplementary material for: Model Evaluation of the Microbial Metabolic Processes in a Hydrogen-Based Membrane Biofilm Reactor for Simultaneous Bromate and Nitrate Reduction
Source: Membranes (Basel). 2022 Aug 11;12(8):774. doi: 10.3390/membranes12080774 (PMC9415787; doi:10.3390/membranes12080774)
Supplement: Supplementary file 1 [file membranes-12-00774-s001.zip › membranes-1847813-supplementary.pdf]

Supplementary Material

Model Evaluation of the Microbial Metabolic Processes in a Hydrogen-Based Membrane Biofilm Reactor for Simultaneous Bromate and Nitrate Reduction

Minmin Jiang [1,2], Yuanyuan Zhang [2], Jie Zhang [2,3,4], Xingru Dai [2], Haixiang Li [1,*], Xuehong Zhang [1,2], Zhichao Wu [4] and Junjian Zheng [2,4,*]

1 Guangxi Key Laboratory of Environmental Pollution Control Theory and Technology, Guilin University of Technology, 319 Yanshan Street, Guilin 541006, China; jiangminmin1015@163.com (M.J.); zhangxuehong@x263.net (X.Z.)
2 College of Life and Environmental Science, Guilin University of Electronic Technology, 1 Jinji Road, Guilin 541004, China; zhangyuanyuan0226@hotmail.com (Y.Z.); jieemma2016@foxmail.com (J.Z.); xingrudai@163.com (X.D.)
3 School of Chemistry and Materials Engineering, Huizhou University, 46 Yanda Road, Huizhou 516007, China
4 State Key Laboratory of Pollution Control and Resource Reuse, School of Environmental Science and Engineering, Tongji University, 1239 Siping Road, Shanghai 200092, China; wuzhichao@tongji.edu.cn
* Correspondence: 2011042@glut.edu.cn (H.L.); zhengjunjianglut@163.com (J.Z.)

Table S1. Short-term operational conditions of influencing factor experiment of the H_2-MBfR.

Short-term series	H_2 pressure (MPa)	Influent NO_3^- (mg/L)	CO_2 pressure (MPa)	Other conditions
H_2 series	0.01-0.08	10	0.012	Influent flowrate=2.0mL/min
NO_3^- series	0.04	1-20	0.012	HRT=4.7
CO_2 series	0.04	10	0.004-0.036	Influent BrO_3^-=1.0mg/L<br>Influent NO_3^-=10mgN/L

Table S2. Start-up and long-term operational conditions of the H_2-MBfR.

Stages	Periods (days)	H_2 pressure (MPa)	CO_2 pressure (MPa)	Influent NO_3^- (mgN/L)	Influent BrO_3^- (mg/L)	Influent flowrate (mL/min)	HRT (h)
Stage1	0~40	0.04	0.012	10	0	1.6/2.0	5.8/4.7
Stage2	41~60	0.04	0.012	10	0	2.0	4.7
Stage3	61~80	0.04	0.012	10	0.1	2.0	4.7
Stage4	81~100	0.04	0.012	10	0.5	2.0	4.7
Stage5	101~140	0.04	0.012	10	1.0	2.0	4.7

Table S3. Stoichiometric and kinetic parameters of BRB related metabolic process for the developed model.

Parameter	Definition	Value	Unit	Source
$f^0_{s,BRB}$	Fraction of electrons for cell synthesis reaction on BRB	0.591	-	Theoretically calculated*
$f^0_{e,BRB}$	Fraction of electrons for energy providing reaction on BRB	0.409	-	Theoretically calculated*
μ_{BRB}	Maximum specific growth rate of BRB	0.85	1/d	Recalibrated
K_{BrO3}	Half maximum rate concentration of BrO_3^- for BRB	0.014	mg/L	Recalibrated

Table S4. Experiments operational parameters for the model development.

Parameters	Symbol	Value	Unit
Total reactor volume	V	$5.6 \cdot 10^{-4}$	m^3
Membrane area	A	$6.33 \cdot 10^{-2}$	m^2
Influent velocity	Q_{in}	2	mL/min
Influent BrO_3^- concentration	S_{BrO3}	0.1-1.0	mg/L

H_2 pressure	P_{H2}	0.01-0.08	MPa
Influent NO_3^- concentration	S_{SNO3}	1-20	mgN/L
CO_2 pressure	P_{CO2}	0.004-0.036	MPa

Table S5. Other pertinent input parameters for model development.

Symbol	Description	Value	Unit	Source
Stoichiometric parameters				
$f^0_{e,DNB}$	Portion of electrons for energy providing reaction of H_2-based denitrification	0.462	-	(Jiang et al., 2020)
$f^0_{s,DNB}$	Portion of electrons for biomass synthesis of DNB	0.538	-	(Jiang et al., 2020)
Y_{HB}	Yield for HB growing on SMP	0.67	mol COD/mol COD	(Henze et al., 2008)
k_1	Fraction of electrons going to biomass production	0.77	-	(Tang et al., 2012)
k_2	Fraction of electrons going to SMP formation	0.05	-	(Tang et al., 2012)
k_3	Fraction of electrons going to EPS formation	0.18	-	(Tang et al., 2012)
f_d	Fraction of biodegradable biomass	0.8	-	(Tang et al., 2012)
Kinetic parameters				
μ_{DNB}	Maximum specific growth rate of DNB	0.57	1/d	Recalibrated
μ_{HB}	Maximum specific growth rate of HB	4	1/d	(Jiang et al., 2020)
K_{H2}^{DNB}	Half maximum rate concentration of H_2 for DNB	0.001	mol H_2/m^3	(Tang et al., 2012)
K_{NO3}^{DNB}	Half maximum rate concentration of NO_3^- for DNB	0.0143	mol N/m^3	(Tang et al., 2012)
K_{CO2}^{DNB}	Half maximum rate concentration of CO_2 for DNB	0.0001	mol CO_2/m^3	(Jiang et al., 2020)
K_{H2}^{BRB}	Half maximum rate concentration of H_2 for BRB	0.001	mol H_2/m^3	(Jiang et al., 2020)
K_{NO3}^{BRB}	Half maximum rate concentration of NO_3^- for BRB	0.0143	mol N/m^3	(Jiang et al., 2020)
K_{CO2}^{BRB}	Half maximum rate concentration of CO_2 for BRB	0.0001	mol CO_2/m^3	(Jiang et al., 2020)
K_{NO3}^{HB}	Half maximum rate concentration of NO_3^- for HB	0.0143	mol N/m^3	(Tang et al., 2012)
K_{SMP}^{HB}	Half maximum rate concentration of SMP for HB	0.156	mol COD/m^3	(Chen et al., 2017)
b_{DNB}	Specific decay rate of DNB	0.05	1/d	(Jiang et al., 2020)
b_{BRB}	Specific decay rate of BRB	0.05	1/d	(Jiang et al., 2020)
b_{HB}	Specific decay rate of HB	0.1	1/d	(Jiang et al., 2020)
k_{hyd}	Hydrolysis rate of EPS	0.22	1/d	(Tang et al., 2012)
Physical parameters				
D_{H2}	Diffusion coefficient of H_2 in water	$4.415 \cdot 10^{-4}$	m^2/d	(Lide, 2012)
D_{NO3}	Diffusion coefficient of NO_3^- in water	$1.643 \cdot 10^{-4}$	m^2/d	(Lide, 2012)
D_{BrO3}	Diffusion coefficient of BrO_3^- in water	$1.281 \cdot 10^{-4}$	m^2/d	(Lide, 2012)
D_{CO2}	Diffusion coefficient of CO_2 in water	$1.650 \cdot 10^{-4}$	m^2/d	(Lide, 2012)
D_{SMP}	Diffusion coefficient of SMP in water	$8.64 \cdot 10^{-5}$	m^2/d	(Lide, 2012)

f_{dif}	Diffusion in biofilm/in water	0.75	-	(Jiang et al., 2020)
H_{H2}	Henry coefficient of H_2	0.019	-	(Jiang et al., 2020)
K_m	H_2 transfer coefficient of HFM	0.189	m/d	Recalibrated

Section S1. Calculation of the stoichiometric coefficients for BRB metabolic process (method (Henze et al., 2008; Jiang et al., 2020)).

The stoichiometric coefficient for BRB metabolic process was theoretically calculated based on bioenergetics. The calculation procedure consists of the following 4 steps.

A. Energy providing reaction (catabolism)

The reaction and energy available from the autotrophic growth with H_2 as electron donor and BrO_3^- as electron acceptor is:

$$\frac{1}{2}H_2 + \frac{1}{6}BrO_3^- = \frac{1}{6}Br^- + \frac{1}{2}H_2O \qquad -138.98\ \text{kJ/eeq} \qquad S1.1$$

$$\Delta G_{cata} = K\Delta G_R = 0.60\times(-138.98) = -83.39\ \text{kJ/eeq} \qquad S1.2$$

where: ΔG_{cata} is th Gibbs energy available for catabolism from 1 eeq of electron donor (kJ/eeq), K is the fraction of energy transfer captured (typically 0.60), ΔG_R is the Gibbs energy released from 1 eeq of electron donor (kJ/eeq), ΔG_R = -138.98 kJ/eeq according to S1.1.

B. Energy needed for cell synthesis (anabolism)

Referring to the references (Henze et al., 2008; Jiang et al., 2020), the energy needed to synthesize biomass from H_2 as electron donor and NO_3^- as nitrogen source was that ΔG_{ana} = 57.702 kJ/eeq.

C. Determin f_e^0 (energy fraction) and f_s^0 (cell synthesis fraction)

$$f_s^0 = \frac{1}{1+\frac{f_e^0}{f_s^0}} = \frac{1}{1+\frac{\Delta G_{ana}}{-\Delta G_{cata}}} = \frac{1}{1+\frac{57.702}{83.39}} = 0.591 \qquad S1.3$$

$$f_e^0 = 1 - f_s^0 = 1 - 0.591 = 0.409 \qquad S1.4$$

D. Solve for the stoichiometric coefficients for all the components with BRB autotrophic growth on H_2

$$(\text{ED reaction}) + (\text{EA reaction}) \times f_e^0 + (\text{Synthesis reaction}) \times f_s^0 \qquad S1.5$$

$$\frac{1}{2}H_2 + \frac{f_s^0}{28}NO_3^- + \frac{f_e^0}{6}BrO_3^- + \left(f_e^0 + \frac{29f_s^0}{28} - 1\right)H^+ + \frac{5f_s^0}{28}CO_2 = \frac{f_e^0}{6}Br^- + \frac{f_s^0}{28}C_5H_7O_2N + \left(\frac{f_e^0}{2} + \frac{11f_s^0}{28}\right)H_2O$$

S1.6

Substitute the values of f_e^0 and f_s^0 into S1.6:

$$0.5H_2 + 0.021NO_3^- + 0.068BrO_3^- + 0.021H^+ + 0.106CO_2 = 0.068Br^- + 0.021C_5H_7O_2N + 0.437H_2O \quad \text{S1.7}$$

References

Chen, X., Liu, Y., Peng, L., Ni, B.-J., 2017. Perchlorate, Nitrate, and Sulfate Reduction in Hydrogen-based Membrane Biofilm Reactor: Model-based Evaluation. Chemical Engineering Journal 316, 82-90. https://doi.org/10.1016/j.cej.2017.01.084.

Henze, M., van Loosdrecht, M.C., Ekama, G.A., Brdjanovic, D., 2008. Biological Wastewater Treatment, IWA publishing, London, UK.

Jiang, M., Zheng, J., Perez-Calleja, P., Picioreanu, C., Lin, H., Zhang, X., Zhang, Y., Li, H., Nerenberg, R., 2020. New Insight into CO_2-mediated Denitrification Process in H_2-based Membrane Biofilm Reactor: An Experimental and Modeling Study. Water Research 184, 116177. https://doi.org/10.1016/j.watres.2020.116177.

Lide, D.R., 2012. CRC handbook of chemistry and physics, CRC Boca Raton, Florida, US.

Tang, Y., Zhao, H., Marcus, A.K., Krajmalnik-Brown, R., Rittmann, B., E, 2012. A steady-state Biofilm Model for Simultaneous Reduction of Nitrate and Perchlorate, Part 1: Model Development and Numerical Solution. Environmental Science & Technology 46(3), 1598-1607. https://doi.org/10.1021/es203129s.
